# A transporter’s doom or destiny: *SLC6A1* in health and disease, novel molecular targets and emerging therapeutic prospects

**DOI:** 10.3389/fnmol.2024.1466694

**Published:** 2024-08-29

**Authors:** Nikita Shah, Ameya S. Kasture, Florian P. Fischer, Harald H. Sitte, Thomas Hummel, Sonja Sucic

**Affiliations:** ^1^Institute of Pharmacology, Medical University of Vienna, Vienna, Austria; ^2^Department of Epileptology and Neurology, RWTH Aachen University, Aachen, Germany; ^3^Hourani Center for Applied Scientific Research, Al-Ahliyya Amman University, Amman, Jordan; ^4^Center for Addiction Research and Science-AddRess, Medical University of Vienna, Vienna, Austria; ^5^Department of Neuroscience and Developmental Biology, University of Vienna, Vienna, Austria

**Keywords:** *SLC6A1*, GABA transporter 1, human disease variants, cellular quality control mechanisms, protein trafficking, epilepsy, intellectual disability, protein misfolding

## Abstract

As the first member of the solute carrier 6 (SLC6) protein family, the γ-aminobutyric acid (GABA) transporter 1 (GAT1, *SLC6A1*), plays a pivotal role in the uptake of GABA from the synaptic cleft into neurons and astrocytes. This process facilitates the subsequent storage of GABA in presynaptic vesicles. The human *SLC6A1* gene is highly susceptible to missense mutations, leading to severe clinical outcomes, such as epilepsy, in the afflicted patients. The molecular mechanisms of *SLC6A1*-associated disorders are discerned to some degree; many *SLC6A1* mutations are now known to impair protein folding, and consequently fail to reach the plasma membrane. Inherently, once inside the endoplasmic reticulum (ER), GAT1 abides by a complex cascade of events that enable efficient intracellular trafficking. This involves association with specialized molecular chaperones responsible for steering the protein folding process, oligomerization, sorting through the Golgi apparatus, and ultimately delivery to the cell surface. The entire process is subject to stringent quality control mechanisms at multiple checkpoints. While the majority of the existing loss-of-function *SLC6A1* variants interfere with folding and membrane targeting, certain mutants retain abundant surface expression. In either scenario, suppressed GAT1 activity disrupts GABAergic neurotransmission, preceding the disease manifestation in individuals harboring these mutations. The nervous system is enthralling and calls for systematic, groundbreaking research efforts to dissect the precise molecular factors associated with the onset of complex neurological disorders, and uncover additional non-canonical therapeutic targets. Recent research has given hope for some of the misfolded *SLC6A1* variants, which can be salvaged by small molecules, i.e., chemical and pharmacological chaperones, acting on multiple upstream targets in the secretory pathway. We here highlight the significance of pharmacochaperoning as a therapeutic strategy for the treatment of *SLC6A1*-related disorders.

## The ABC’s of *SLC6A1*: basic physiology and structural features

The inhibitory neurotransmitter γ-aminobutyric acid (GABA) is essential for the regulation and function of various cortical and sub-cortical circuits. The right balance between excitation and inhibition in neural circuits is physiologically absolutely vital, and can lead to seizures when imbalanced (Treiman, 2001). GABA, released from the GABAergic neurons, is rapidly retrieved by the designated GABA transporters (GATs), expressed in GABAergic neurons and glia (Fattorini et al., 2020). Among the four GAT isoforms belonging to the solute carrier 6 (*SLC6*) family, GAT1 (*SLC6A1*) is the major GABA transporter in the central nervous system. It is responsible for clearing GABA from the extracellular space, thus establishing the basis for GABAergic signaling that terminates neurotransmission. The translocation of GABA via GAT1 depends on an inward electrochemical gradient, involving a co-transport of Na^+^ and Cl^−^ across the membrane (Scimemi, 2014). The GAT1 has 12 transmembrane domains in helical stretches, intracellular amino (N)- and carboxyl (C)-termini, and three glycosylation sites in its large second extracellular loop (Bennett and Kanner, 1997; Motiwala et al., 2022). Fluorescence resonance energy transfer (FRET) studies in HEK293 cells revealed that GAT1 exists as an assembly of multiple monomers (Schmid et al., 2001), with each moiety capable of independent GABA uptake, as shown in *Xenopus laevis* oocytes (Soragna et al., 2005). Oligomerization also serves as a prerequisite for the Sec24D-dependent concentrative export of GAT1 from the endoplasmic reticulum (ER) compartment (Farhan et al., 2006).

## Cellular mechanisms of GAT1 synthesis and delivery to the plasmalemma

**Figure 1:**
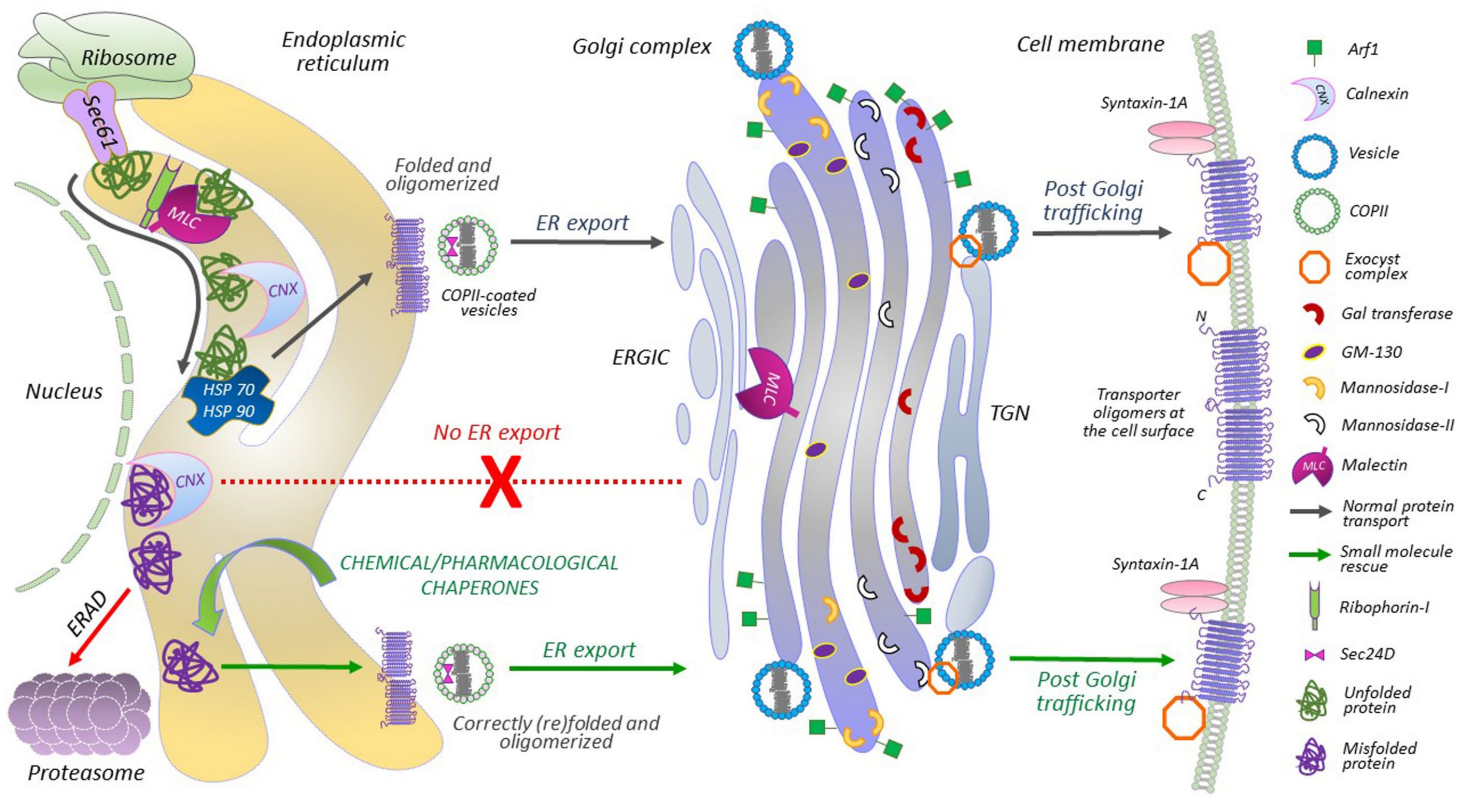
A detailed portrayal of GAT1 protein folding and membrane trafficking. Ribosome-translated nascent polypeptides are inserted into the ER membrane via the Sec61 translocon. The translocon provides a channel that shields the hydrophobic residues of the nascent chain, identifies transmembrane domains and hydrophobic signal peptides, and facilitates their lateral exit into the lipid bilayer. After lateral exit and N-linked glycosylation, transporters undergo calnexin/calreticulin cycles. Malectin, associated with ribophorin-I, also interacts with folding intermediates before the calnexin cycles begin, at sites distinct from the calnexin binding site in the ER and Golgi. HSP relay binds to the folding protein from the cytoplasmic side. Misfolded proteins or folding-intermediates, bound to chaperones, adhere to the ER-associated degradation (ERAD) pathway. Disengagement of HSP relay allows the engagement of COPII components with the natively folded oligomerized transporters, i.e., Sec24D interacts with the ER export motif located on the C-terminal of GAT1. From the ER-Golgi intermediate complex (ERGIC), GAT1 cargo is further transported to and through the Golgi complex. Exocyst and syntaxin 1A are recruited for proper targeting and insertion of GAT1 oligomers at the plasma membrane. Chemical and pharmacological chaperones can correct the folding of ER-retained/misfolded transporter mutants and rescue their expression (and activity) at the cell membrane.

Being a transmembrane protein, GAT1 is largely hydrophobic, and its biogenesis machinery is similar to other secretory proteins, i.e., it comprises a series of steps dependent on the recognition of a hydrophobic peptide segment of the newly synthesized protein via the signal recognition particle (SRP). This recognition simultaneously slows down the translation process in the ribosomes (Korkhov et al., 2008). Subsequently, the hydrophobic peptide is directed towards the ER for insertion into the ER membrane via the Sec61 translocon complex, followed by the resumption of the translation process by ribosomes (Figure 1). As the translation of eukaryotic proteins is rather slow, protein folding takes place co-translationally (Balchin et al., 2016); i.e., protein synthesis and the insertion of peptide segments into the ER membrane occur concomitantly. During folding, the proteins undergo post-translational modifications, such as N-glycosylation and disulfide bond formation (Caramelo and Parodi, 2015). Molecular chaperone systems, like lectin-chaperones (e.g., calnexin, calreticulin, and malectin) and ATP-dependent molecular chaperones (e.g., heat shock proteins, HSPs) assist the folding process. This occurs in a highly coordinated manner, such that the glycoprotein undergoes numerous calnexin cycles, i.e., re-association of the protein with calnexin to achieve a natively folded structure, until it is no longer recognizable by calnexin, which in turn, prepares the protein cargo for ER exit (Booth and High, 2004).

The ER of eukaryotic cells is a very crowded place: the folding of large proteins can be inefficient and result in folding intermediates and misfolded proteins (Figure 1). Missense mutations, in, e.g., *SLC6* transporters, generally complicate this process even further. Proteins that fail to achieve a native state are removed from the system via ER-associated degradation (ERAD; Booth and High, 2004). The natively folded GAT1 forms homo-oligomers before exiting the ER (Scholze et al., 2002). Oligomeric GAT1 recruits Sec24D [a component of the coat protein complex II (COPII)] for concentrative ER export, which plays a key role in determining GAT1 levels at the plasma membrane (Farhan et al., 2007). Mutating the ER export motif on the C-terminus of GAT1, impedes its interaction with Sec24D, and exerts a dominant negative effect on wild-type GAT1 expression at the cell surface (Farhan et al., 2007). Similar effects were recently observed for a related SLC6 transporter, creatine transporter 1 (CRT1, *SLC6A8*), upon elimination of its N-terminal ER export motif (Ün et al., 2024).

COPII vesicles bud off the ER to form membrane-like fused structures termed the ER-Golgi intermediate compartment (ERGIC), from which GAT1 is further channeled toward the Golgi via a specific C-terminal motif (Farhan et al., 2008) for intra-Golgi trafficking (Figure 1). Additional post-translational modifications take place in the Golgi, which remove three residual mannose residues and adds complex molecules, like N-acetylglucosamines, fucose, galactose and sialic acid residues, which are required for mature glycosylation and priming the protein for the plasma membrane (Mohan et al., 2023). In essence, the proteins traverse through the cisternal membranes of the Golgi from *cis* to *trans* compartments, which are the key functional elements of the Golgi, with differential resident processing enzymes required for glycosylation (Jackson, 2009). The trafficking of secretory proteins from the *trans*-Golgi compartment to membrane surfaces remains poorly understood. Tubular pleomorphic structures budding from the *trans*-Golgi compartment are believed to be the carriers that transport secretory proteins directly to the plasma membrane (Stalder and Gershlick, 2020). The exocyst complex, however, is a well-known requirement for the regulated membrane insertion of the GAT1 (Farhan et al., 2004). The last three amino acids of GAT1 (AYI) interact with the exocyst (Farhan et al., 2004). The removal or substitution of these residues affects the membrane insertion of the transporters in cultured cells (Farhan et al., 2004).

Surface GAT1 levels are also highly orchestrated by other proteins, including the soluble N-ethylmaleimide-sensitive factor (NSF) attachment protein receptors (SNAREs), syntaxin-1A and synaptosomal-associated protein 25 (SNAP-25; Quick et al., 2004; Quick, 2006). Syntaxin-1A interacts with the N-terminus of GAT1 and increases its surface expression (Deken et al., 2000). Conversely, syntaxin-1A also acts as a negative regulator of GABA translocation, as the binding of syntaxin-1A to the N-terminus of GAT1 inhibits the flux, which can be reversed by GABA, due to its dissociation from syntaxin-1A (Quick, 2006). Thus, syntaxin-1A plays a dual role in tuning extracellular neurotransmitter levels by regulating GAT1 surface expression via trafficking, and by modulating GABA transport. Syntaxin-1A is also involved in the docking and fusion of GABA-containing vesicles, suggesting that it regulates GABA release and reuptake. Interestingly, missense mutations in the gene encoding syntaxin-1A are also associated to epilepsy (Luppe et al., 2023).

## *SLC6A1* loss-of-function pathogenic variants

**Figure 2:**
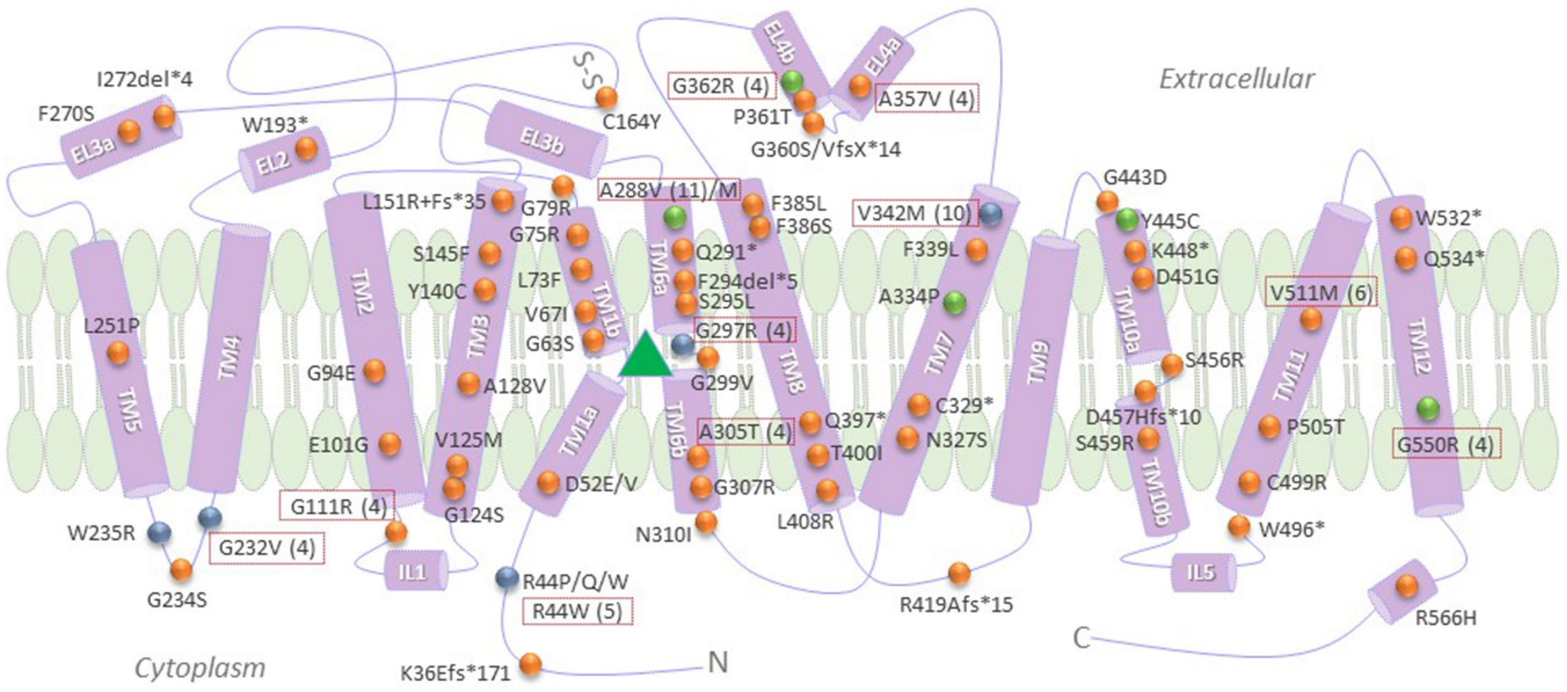
Pathogenic SLC6A1 variants, categorized according to known molecular defects, frequency and response to pharmacochaperone rescue. Recurrent mutations are shown in dotted red boxes, with the number of the reported cases indicated in brackets. Green spheres denote those variants that were shown to be amenable to rescue by pharmacological and/or chemical chaperones, to date (Kasture et al., 2023). Blue spheres indicate loss-of-function mutations that did not impair membrane trafficking properties of GAT1 (Kasture et al., 2023). Orange spheres denote any other reported mutations with a loss-of-function phenotype, many of which still necessitate further investigation into their specific molecular mechanism/s of disease. The green triangle represents the binding pocket for GABA, i.e., the region encompassing TMs 3 and 8 and the hinge domains of TMs 1 and 6. TM, transmembrane domains; Fs, frameshift; “*” and “del,” termination codon and deletion mutations, respectively; N and C, amino and carboxyl termini, respectively.

Mutations in the *SLC6A1* gene, which encodes GAT1, have been associated with a wide range of neurodevelopmental disorders, such as autism, intellectual disability, and epilepsy syndromes (Fischer et al., 2022). The loss-of-function phenotype imparted by the mutations are attributed to single nucleotide variants or missense mutations, frameshift (fs)-, termination codon-and splice site-mutations (Figure 2). The *SLC6A1* gene is more sensitive to disruption by missense mutations than many other genes (Silva et al., 2024). Recurrent missense mutations (red boxes in Figure 2) are located at hypermutable CpG sites (Silva et al., 2024). These disease-linked missense mutations can be broadly classified into two groups, based on defective protein folding/trafficking or functional activity of the resulting GATs (Figure 2). Mutations that affect GABA transport, but not trafficking, typically impair the substrate binding site or its translocation pathway (blue spheres in Figure 2). In contrast, folding/trafficking-deficient variants are retained at the ER, but may exhibit substrate binding affinity similar to the wild-type protein (Figure 2, orange spheres), and are amenable to rescue by pharmacological means (green spheres in Figure 2).

## Digging deep in the ER: hunting for novel molecular and drug targets

SLC6 transporters undergo numerous conformational changes to reduce the free energy of folding and to attain a natively folded state. This is assisted by an elegant cascade of molecular chaperones and quality control mechanisms, described above. Point mutations in *SLC6A1* often disrupt the habitual folding trajectory of the transporter, trapping it in a misfolded state at the ER level. ER-resident chaperones play a crucial role in assisting such transporters (Korkhov et al., 2008; Kasture et al., 2016). The lectin-based luminal chaperone calnexin (Figure 1), can act as a “folding sensor” (Ou et al., 1993); e.g., it was shown to bind an oligomerization-deficient E101D-GAT1 mutant with markedly higher affinity than the wild-type (Korkhov et al., 2008). Similar observations were made for misfolded mutants in the transporters for dopamine (DAT, *SLC6*A3) and serotonin (SERT, *SLC6*A4; El-Kasaby et al., 2010; Koban et al., 2015; Kasture et al., 2016).

Malectin, a stress-responsive membrane-anchored-ER-resident lectin is responsible for the quality control of glycoproteins (Schallus et al., 2008, 2010; Qin et al., 2012; Feng et al., 2022). Malectin preferentially binds to misfolded proteins and inhibits their ER export (Qin et al., 2012). It does not compete with calnexin for substrate binding, suggesting an additional layer of lectin-based quality control for glycoproteins (Galli et al., 2011). Considering that malectin does not have an ER-retention peptide of its own, it associates with ribophorin-I for selective recognition of misfolded glycoproteins in the ER. Upon suppression of ribophorin-I and under ER stress conditions, malectin diffuses into Golgi membranes (Figure 1; Yang et al., 2018). It is tempting to envision that relaxing or modifying lectin-based chaperones might enhance the trafficking of misfolded GAT1 mutants.

Throughout the folding process, the cytosolic segments of *SLC6* transporters are monitored by the HSP relay; i.e., SERT harbors a HSP70-binding motif, RLIIT, on its C-terminus (El-Kasaby et al., 2014; Koban et al., 2015). SERT specifically recruits HSP70-1A at the ER (El-Kasaby et al., 2014). A chaperone-COPII-exchange model for *SLC6* transporters postulates that detachment of calnexin from the transporter is essential for oligomerization, and disengagement of HSP70 (Figure 1) is required prior to recruiting COPII components (Chiba et al., 2014). Folding-deficient transporters that remain bound to the chaperones, are eventually subjected to ERAD (Figure 1). Hence, relaxing the quality control at the ER may contribute to the mechanisms of rescue of ER-trapped transporters (Kasture et al., 2017). In both, HEK293 cells and *Drosophila melanogaster*, we demonstrated the potential of a HSP70 inhibitor (pifithrin-μ) in the rescue of some infantile parkinsonism-linked misfolded DAT variants (Mazhar Asjad et al., 2017). Similarly, a HSP 90 blocker (17-DMAG) promoted the expression and activity of synthetic folding-impaired SERT mutants in HEK293 cells (El-Kasaby et al., 2014). To date, the treatment of several GAT1 variants tested with blockers of HSP70 (pifithrin-μ and YM-08) or HSP90 (17-DMAG and NVP-HSP990) failed to yield any appreciable effect (Kasture et al., 2023). Thus, despite certain similarities, individual mutants of *SLC6* transporters can respond differently and selectively to particular pharmacological manipulations.

There are numerous direct and indirect ER-targeting strategies for delivering small molecules to the ER (Shi et al., 2021). For instance, the Lys-Asp-Glu-Leu (KDEL) receptor system can be utilized to deliver drugs to the ER organelle. The KDEL peptide fused to 4-phenylbutyric acid (4-PBA) was recently used to target the chemical chaperone to the ER (Azoulay-Ginsburg et al., 2021) in a *Drosophila* model of amyotrophic lateral sclerosis (ALS), where the targeted delivery of 4-PBA significantly reduced retinal degradation at minimal drug concentrations (Azoulay-Ginsburg et al., 2021). Such targeted ER delivery techniques might prove particularly favorable, in that they substantially improve the bioavailability of small molecules.

## Pursuing Golgi dynamics: yet another (complex) entity to embark upon?

The Golgi complex is a highly compact and organized structure, maintained by different regulators such as the cytoskeleton, Golgi structural proteins, motor proteins, kinases and small guanosine triphosphatases (GTPases). The key small GTPase, ADP-ribosylation factor 1 (Arf1) controls Golgi dynamics and function, in a cell matrix adhesion-dependent manner, i.e., in the anchorage dependent cells (Singh et al., 2018; Rajeshwari et al., 2023). The ER and Golgi must operate in perfect tandem, in order to process the enormous protein load *en route* to the plasma membrane. Golgi organization is shaped by the ER machinery, wherein ER stress can disrupt the Golgi structure, a concept known as Golgi fragmentation (Nakagomi et al., 2008). Disrupted Golgi dynamics can hence pose momentous bearing in disease development. An intact Golgi complex has the potential to sense the stress and death cues from the upstream intracellular “trafficking-related players.” The GAT1 itself strides along the Golgi path. Since many epilepsy-linked GAT1 variants are stalled in the ER compartment (Kasture et al., 2023), it is feasible that the ensuing ER stress propagates, with an aftermath on Golgi dynamics. A recent study linked Golgi fragmentation with seizures in epileptic patients, as well as in *in vivo* studies in rats (Skupien-Jaroszek et al., 2023).

## The long and winding road from molecular mechanisms of disease to the rescue

The molecular chaperone system in cells facilitates proteins in achieving their thermodynamically stable and natively folded states. In the instance of misfolded GAT1 and other SLC6 transporters, this internal chaperone machinery falls short. However, folding-deficient mutants can be rescued by small molecules, i.e., chemical and pharmacological chaperones. Examples of compounds that have been effective in improving the expression and activity of such SLC6 variants, either *in vitro* or *in vivo*, are listed in Table 1. Chemical chaperones [e.g., glycerol, 4-PBA and dimethyl sulfoxide (DMSO)] are non-specific, and act on a wide range of proteins to promote their folding and delivery to the cell surface. Conversely, pharmacological chaperones (pharmacochaperones) display substrate specificity and bind and stabilize the target protein (Loo and Clarke, 2007). The folding trajectory of SLC6 transporters progresses through the inward-facing conformation of the transport cycle (Sucic et al., 2016; Kasture et al., 2017). Drugs that bind and stabilize the inward-facing state of DAT and SERT can thus restore the folding/trafficking of these transporters (Kasture et al., 2016; Bhat et al., 2017; Mazhar Asjad et al., 2017). One such compound is noribogaine, which effectively restored the surface expression of misfolded transporters in cell lines and in living fruit flies (Kasture et al., 2016; Bhat et al., 2017; Mazhar Asjad et al., 2017). Tiagabine, a clinically approved antiseizure drug, locks GAT1 in its inward-open conformational state (Motiwala et al., 2022). In HEK293 cells expressing the GAT1 epilepsy-linked A288V variant (one of the most commonly recurrent mutations identified in patients; cf., Figure 2), we found a rather modest increase in uptake upon exposure to tiagabine. Surprisingly, in transgenic flies, the same treatment yielded significant increases in the surface expression of both wild-type GAT1 and A288V. Despite this, tiagabine treatment failed to reduce seizures in flies harboring the A288V mutation (Kasture et al., 2023). However, unsurpassed rescue was observed upon treatment with the chemical chaperone 4-PBA (Kasture et al., 2023). In addition to the role of 4-PBA in preventing the aggregation of misfolded proteins, it is also known to reduce ER stress (Kubota et al., 2006) and downregulate HSP70 activity (Rubenstein and Lyons, 2001). Since this chaperone modulates a wide range of activities at the cellular and molecular levels, it likely enhances the trafficking process in more ways than one. Previously, we showed that 4-PBA rescues folding-deficient CRT1 mutants, associated with the debilitating creatine transporter deficiency syndrome (El-Kasaby et al., 2019; Farr et al., 2020). SLC6 transporters are receptive to assimilating different substrates and co-substrates, allowing for flexibility, thus making them excellent drug targets (Bhat et al., 2021).

**Table 1:** Small molecules demonstrating successful restoration of cell surface expression and uptake activity of misfolded SLC6 transporter variants.

SLC6 member	Compounds with rescue potential	Misfolded variants	*In vitro/in vivo* study type	References
hDAT (*SLC6A3*)	Noribogaine and pifithrin-μ	V158F, G327R, L368Q	Cell culture; humanized *Drosophila*	Mazhar Asjad et al. (2017)
	Pifithrin-μ	L368Q	Patient-derived iPSCs	Ng et al. (2021)
	Bupropion	A314V, R445C	Cell culture	Beerepoot et al. (2016)
	Ibogaine analog 9b	A314V, G386R, P395L, R445C, R521W, P554L	Cell culture; humanized *Drosophila*	Bhat et al. (2021)
hSERT (*SLC6A4*)	Ibogaine and DMSO	RI(607,608)AA, RII(607-609)AAA	Cell culture	El-Kasaby et al. (2010)
	Noribogaine, pifithrin-μ, and 17-DMAG	PG(601,602)AA, F604Q, RI(607,608)AA	Cell culture	El-Kasaby et al. (2014)
	Phenethylamine (PAL)-287, −1,045, −1,046	PG(601,602)AA	Cell culture	Bhat et al. (2017)
	Ibogaine, noribogaine, ibogaine analogs 3b, 3c, 4c, 9a, 9b, 9c, 9d, PAL-287, −1,045, −1,046 and bupropion	PG(601,602)AA	Cell culture	Bhat et al. (2021)
	Trazodone, nefazodone, and vilazodone	PG(601,602)AA	Cell culture; humanized *Drosophila*	El-Kasaby et al. (2024)
hCRT1 (*SLC6A8*)	4-PBA	R391W, A404P, G424D, V539I, P544L, P554L	Cell culture	El-Kasaby et al. (2019)
hGlyT2 (*SLC6A5*)	4-PBA	Rescue of WT GlyT2 from the dominant negative S512R variant	Primary neurons	Arribas-González et al. (2015)
hGAT1 (*SLC6A1*)	4-PBA, liothyronine, and tiagabine	A288V, A334P, G362R, Y445C	Cell culture; humanized *Drosophila*	Kasture et al. (2023)

## Shaken but not stirred: acumens from recent studies in humanized flies

Targeted trafficking of transporter proteins is essential in both asymmetric cells like neurons, as well as in cells with complex morphologies, such as astrocytes. *In vivo* models allow us to study protein folding and trafficking in a physiological context. Due to their short life cycle, extensive genetic resources, ease of maintenance, and cost-effectiveness, *Drosophila melanogaster* has become a widely used animal model in studies of various aspects of neurobiology and neurological disorders (Kasture et al., 2018; Fischer et al., 2022, 2023). About 83% of all SLC superfamily of transporters are conserved between humans and flies (Ceder and Fredriksson, 2022). Most of the genes involved in the folding and quality control of proteins are also conserved across species. As a matter of fact, the HSP genes were first discovered in *Drosophila* (Ritossa, 1962). Mendelian or monogenic diseases are widely studied in flies. Various aspects of human proteins can be studied in a temporal and spatial manner by expressing them in fruit flies (Rubin and Spradling, 1982; Brand and Perrimon, 1993; Gratz et al., 2015; Mazhar Asjad et al., 2017; Kasture et al., 2019). We and others have studied disease-relevant human *SLC6* transporters and ion channels in fruit flies (Kasture et al., 2016, 2017, 2018, 2019; Sucic et al., 2016; Campbell et al., 2019; Hussain et al., 2024). *Drosophila* has a single GABA transporter (dGAT), expressed primarily in astrocytes (Stork et al., 2014). In addition to expressing dGAT, astrocytes also express a transporter for glutamate (Soustelle et al., 2002), thus regulating both extracellular GABA and glutamate levels. Knockout of the gene encoding dGAT results in embryonic lethality, while astrocytic knockdown induces locomotor defects in larvae and adult flies (Stork et al., 2014). A viable hypomorphic dGAT mutant, with a truncated N-terminus showed markedly reduced expression, increased sleep duration, but reduced sleep latency (Chaturvedi et al., 2022). Membrane expression of dGAT in astrocytes is highly dynamic and regulated by metabotropic GABA receptors (Muthukumar et al., 2014). Increased intracellular Ca^2+^ concentrations in astrocytes lead to rapid endocytosis of dGAT, which results in suppression of neuronal activity (Zhang et al., 2017).

To understand the *in vivo* aspects of GAT1-linked disorders, we generated transgenic flies expressing YFP-tagged A288V-GAT1. These flies expressed both, human GAT1 carrying the A288V mutation, as well as the endogenous dGAT, and presented no recognizable phenotypic defects. The GAT1-A288V variant was trapped in the ER of transfected HEK293 cells, and was comparably contained within the ER compartment in A288V fly astrocytes (Kasture et al., 2023). When expressed in GABAergic neurons, A288V was restricted to the cell bodies (which contain the ER), while the wild-type GAT1 appeared to have a widespread expression pattern (Kasture et al., 2023). The dGAT was shown to modulate seizure behavior in flies (Muthukumar et al., 2014; Mazaud et al., 2019). Accordingly, we observed heat-induced seizures in A288V flies, recapitulating the clinical manifestations in people carrying the A288V mutation (Kasture et al., 2023). Interestingly, knockdown of GAT1 did not trigger heat-induced seizures in flies. In HEK293 cells, treatment with 4-PBA, liothyronine and tiagabine partly restored GABA uptake by A288V mutant. Our earlier studies on DAT taught us that even marginal rescue by pharmacological compounds *in vitro* can have profound physiological effects in *in vivo* systems (Kasture et al., 2016; Mazhar Asjad et al., 2017). In *Drosophila*, treatment with 4-PBA, liothyronine and tiagabine significantly improved the astrocytic expression of A288V in the antennal lobe neuropile of the adult fly brain, mimicking the wild-type GAT1 expression pattern and reduced seizure-like activity (Kasture et al., 2023), illuminating the pharmacochaperoning potential of these drugs, or congeners thereof, *in vivo*. Transgenic flies provide an efficient platform for exploring individual GAT1 pathogenic variants *in vivo*, and most importantly, testing the rescue capacity of various small molecules by, e.g., pharmacochaperoning.

## The existing treatment options for *SLC6A1*-related disorders

At present, there is no specific therapy available to target the underlying pathophysiologic mechanisms of *SLC6A1*-related disorders. Standard treatment is, hence, suboptimal and based on the presenting epilepsy syndrome, rather than the pertinent genetic etiology. Typically, broad spectrum antiseizure medications (ASMs) are used to manage the symptoms. A selection of the most widely used ASMs, in the context of *SLC6A1*-linked conditions, is listed in Table 2. In 2018, Johannesen and colleagues described a cohort of 34 patients harboring mutations in the *SLC6A1* gene, of which 31 had epilepsy (Johannesen et al., 2018). In this cohort, the most common seizure types were absence, myoclonic and atonic seizures. Around half of the patients were diagnosed with epilepsy accompanied with myoclonic-atonic seizures (EMAtS). Two thirds of the patients became seizure free under treatment with ASMs. Ten of these patients took valproic acid either as monotherapy or in combination with clonazepam, lamotrigine or levetiracetam. The remaining individuals became seizure free under treatment with ethosuximide, levetiracetam, lamotrigine or clonazepam, alone or in combination. Vagus nerve stimulation was attempted in one patient with Lennox–Gastaut syndrome without any beneficial effect. Importantly, the authors noted that there was no correlation between seizure control and cognitive outcome. In a recent case report, two further patients with isolated absence seizures were described (Caputo et al., 2023). Both patients became seizure free on valproic acid alone, or in combination with ethosuximide, respectively. Several further case reports described sound responses to valproic acid (Posar and Visconti, 2019; Poliquin et al., 2021; Mermer et al., 2022; Mori et al., 2023; Piniella et al., 2023). The favorable effect of valproic acid in *SLC6A1*-related disorders is not surprising, since it is known to modulate the GABAergic system by increasing the synthesis of GABA and by decreasing its degradation (Chateauvieux et al., 2010).

**Table 2:** A list of commonly used ASMs in the treatment of patients with *SLC6A1*-related disorders.

ASM	Mechanism(s) of action	References
Clobazam	Allosteric modulation of GABA_A_ receptors	Carvill et al. (2015), Kassabian et al. (2023), Piniella et al. (2023), Trivisano et al. (2023)
Clonazepam	Allosteric modulation of GABA_A_ receptors	Carvill et al. (2015), Johannesen et al. (2018), Trivisano et al. (2023)
Ethosuximide	Blockade of calcium channels (T-type)	Carvill et al. (2015), Johannesen et al. (2018), Caputo et al. (2023), Kassabian et al. (2023), Trivisano et al. (2023)
Lamotrigine	Blockade of voltage-gated sodium channels	Carvill et al. (2015), Johannesen et al. (2018), Cai et al. (2019), Kassabian et al. (2023), Mori et al. (2023), Trivisano et al. (2023)
Levetiracetam	Modulation of synaptic vesicle protein 2A (SV2A)	Carvill et al. (2015), Johannesen et al. (2018), Mermer et al. (2022), Kassabian et al. (2023), Trivisano et al. (2023)
Topiramate	Allosteric modulation of GABA_A_ receptors, blockade of AMPA receptors, blockade of voltage-gated sodium channels, inhibition of carbonic anhydrase	Carvill et al. (2015), Johannesen et al. (2018), Kassabian et al. (2023)
Valproic acid	Blockade of voltage-gated sodium channels, blockade of calcium channels (T-type), activation of glutamic acid decarboxylase, inhibition of GABA transaminase	Carvill et al. (2015), Johannesen et al. (2018), Cai et al. (2019), Posar and Visconti (2019), Kahen et al. (2021), Mermer et al. (2022), Caputo et al. (2023), Trivisano et al. (2023), Kassabian et al. (2023), Mori et al. (2023), Piniella et al. (2023), Caraballo et al. (2024)

Beside ASMs, dietary approaches have also been reported as potentially effective treatment options for patients with *SLC6A1*-related disorders. For instance, one variant carrier with EMAtS showed an excellent response to a ketogenic diet. The authors described a virtual cessation of seizures after the diet had been started (Palmer et al., 2016). The ketogenic diet was also described as partially effective in another *SLC6A1* variant carrier in a previous cohort (Carvill et al., 2015). Notably, the ketogenic diet is generally considered a highly effective treatment option for patients with EMAtS (Oguni et al., 2002; Caraballo et al., 2006; Bergqvist, 2012). As most studies focus on the pediatric population, information on the long-term outcome of patients with *SLC6A1*-related disorders is relatively limited. Interestingly, a recent study described the phenotypic spectrum of 15 adult patients with *SLC6A1*-related disorders (Johannesen et al., 2023). In this cohort, epilepsy was prevalent in 11/15 patients and the most common seizure types were absence, myoclonic, atonic and bilateral tonic–clonic seizures. Unfortunately, only four patients became seizure-free. These patients were administered either lamotrigine, valproic acid or a combination of the two drugs.

The high rate of refractory seizures further highlights the vast and unmet clinical need for the development of more efficacious treatments for intractable pediatric conditions, such as *SLC6A1*-triggered epilepsy. A current UK-based pilot clinical trial [NCT05437393; Children’s Adaptive Deep brain stimulation for Epilepsy Trial (CADET)]—is investigating the benefits of a medical device surgically fitted onto a patient’s skull and attached to electrodes which implement deep brain stimulation (DBS). Recently, DBS applied on a teenager suffering from life-threatening epilepsy (due to a mutation in the voltage-gated sodium channel beta subunit 1, SCN1B), ensued an astonishing 80% reduction in both seizure frequency and severity. DBS targeting the thalamic region specifically is known to be helpful in managing refractory seizures irresponsive to regular pharmacological interventions (Fisher, 2023). Since GAT1 shows abundant expression in the thalamus, and normal functioning of the thalamic GAT1 is essential for controlling synchronous spike–and–wave discharges (Cope et al., 2009), it is conceivable that DBS holds potential in handling seizures originating from GAT1 mutations. In light of our recent work (Kasture et al., 2023), it is worth pointing out that an ongoing clinical trial (NCT04937062) focuses on the use of glycerol phenylbutyrate (Ravicti) in patients with *SLC6A1-*and syntaxin-binding protein 1 (STXBP1)-associated developmental and epileptic encephalopathy.

## Summary

In the era of genome sequencing, the identification of monogenic disease-associated mutations is surging worldwide. *SLC6A1* epitomizes a gene especially prone to missense mutations, some of which happen to occur even more frequently due to hypermutable CpG sites (Silva et al., 2024). The current clinical management of *SLC6A1* disorders is far from impeccable. There has been a progressive collective effort, by our group and others, to seek new and more effective pharmacological (and other) means in the treatment of SLC6-related pathologies. The pharmacochaperoning approach, in particular, proved exceptionally advantageous in rectifying protein folding and activity of dozens of pathogenic variants among SLC6 family members (Beerepoot et al., 2016; Kasture et al., 2016, 2023; Bhat et al., 2017, 2021, 2023; Mazhar Asjad et al., 2017; El-Kasaby et al., 2019). 4-PBA, which is FDA-approved for the treatment of urea cycle disorders since the 1980s, has shown great merit in recouping disease variants of hCRT1 (El-Kasaby et al., 2019) and hGAT1 (Kasture et al., 2023). Thorough studies of the molecular machineries in the ER and other organelles, along with the adjoined endogenous quality control processes, ought to disclose prominent new drug targets.

## Glossary

17-DMAG: 17-Dimethylaminoethylamino-17-demethoxygeldanamycin
4-PBA: 4-Phenylbutyric acid
ALS: Amyotrophic lateral sclerosis
Arf1: ADP-ribosylation factor1
ASM: Antiseizure medication
COPII: Coat protein complex II
CRT1: Creatine transporter-1
DAT: Dopamine transporter
DMSO: Dimethyl sulfoxide
EMAtS: Epilepsy with myoclonic-atonic seizures
ER: Endoplasmic reticulum
ERAD: ER-associated degradation
ERGIC: ER-Golgi intermediate compartment
FRET: Fluorescence resonance energy transfer
GABA: γ Aminobutyric acid
GAT1: GABA transporter-1
GTPases: Guanosine triphosphatases
HSP: Heat shock protein
iPSC: Induced pluripotent stem cells
KDEL: Lys-Asp-Glu-Leu
NSF: N-ethylmaleimide-sensitive factor
QC: Quality control
SERT: Serotonin transporter
*SLC6*: Solute carrier 6
SNAP-25: Synaptosomal-associated protein 25
SNARE: Soluble N-ethylmaleimide-sensitive factor attachment protein receptor
SRP: Signal recognition particle
SV2A: Synaptic vesicle protein 2A
